# Evaluation of screening for nasopharyngeal carcinoma: trial design using Markov chain models

**DOI:** 10.1038/sj.bjc.6690301

**Published:** 1999-04

**Authors:** H H Chen, T C Prevost, S W Duffy

**Affiliations:** Graduate Institute of Epidemiology, College of Public Health, Taiwan University, 19 Su-Chou Rd, Taipei, Taiwan; MRC Biostatistics Unit, Institute of Public Health, University Forvie Site, Robinson Way, Cambridge, CB2 2SR, UK

## Abstract

In this paper, we develop a Markov chain model to estimate parameters pertaining to the natural history of nasopharyngeal carcinoma (NPC). The model is of progression from no disease to Epstein–Barr virus (EBV) infection, preclinical screen-detectable tumour and clinical tumour. We derive tentative estimates of the parameters of the model, based on limited published data, to assess the efficacy of serum screening in conjunction with clinical assessment (indirect mirror examination for NPC), for example the average duration of the preclinical screen-detectable phase is estimated as 3.1 years. We further apply these parameters to a hypothetical screening trial in the Hong Kong population to assess the efficacy of serum screening with clinical assessment by different combinations of screening regime. Results suggest: (1) there is no substantial difference between 3-yearly and 6-yearly serum screening; and (2) within the same serum screening regime annual and 3-yearly clinical assessment can prevent 33% and 28% of deaths from NPC respectively. Prediction of deaths and surrogate end points can be used to estimate the required sample size and duration for designing a randomized trial of screening for NPC. Based on these findings and power projections, we suggest a design for a randomized trial in a high incidence area such as Hong Kong. © 1999 Cancer Research Campaign


*British Journal of Cancer* (1999) **79**(11/12), 1894–1900
© 1999 Cancer Research Campaign
Article no. bjoc.1998.0301

### Evaluation of screening for nasopharyngeal carcinoma: trial design using Markov chain models

**HH Chen[1], TC Prevost[2] and SW Duffy[2]**

[1]Graduate Institute of Epidemiology, College of Public Health, Taiwan University, 19 Su-Chou Rd., Taipei, Taiwan; [2]MRC Biostatistics Unit, Institute of Public Health, University Forvie Site, Robinson Way, Cambridge CB2 2SR, UK

**Summary** In this paper, we develop a Markov chain model to estimate parameters pertaining to the natural history of nasopharyngeal carcinoma (NPC). The model is of progression from no disease to Epstein–Barr virus (EBV) infection, preclinical screen-detectable tumour and clinical tumour. We derive tentative estimates of the parameters of the model, based on limited published data, to assess the efficacy of serum screening in conjunction with clinical assessment (indirect mirror examination for NPC), for example the average duration of the preclinical screen-detectable phase is estimated as 3.1 years. We further apply these parameters to a hypothetical screening trial in the Hong Kong population to assess the efficacy of serum screening with clinical assessment by different combinations of screening regime. Results suggest: (1) there is no substantial difference between 3-yearly and 6-yearly serum screening; and (2) within the same serum screening regime annual and 3-yearly clinical assessment can prevent 33% and 28% of deaths from NPC respectively. Prediction of deaths and surrogate end points can be used to estimate the required sample size and duration for designing a randomized trial of screening for NPC. Based on these findings and power projections, we suggest a design for a randomized trial in a high incidence area such as Hong Kong.

**Keywords:**

High age-standardized incidence rates of nasopharyngeal carcinoma (NPC), of the order of 15–30 per 100 000, have been reported for several populations including southern China, Hong Kong, Taiwan, Singapore, and Eskimos in Alaska, Canada and Greenland (Lanier et al, 1980; Zeng et al, 1985; Muir et al, 1987; Chen et al, 1988; Lee et al, 1988; Sasco, 1991). In these areas, NPC is a major cause of cancer death. Since research has shown that 5-year survival for stage I is 80% compared to only 15% for stage IV (Sham and Choy, 1990), it could be of considerable benefit if tumours were detected at an early stage via a screening regime. However, the ability of screening to identify cases in the sub-clinical period is dependent on the evolution of the tumour from biological onset to the manifestation of clinical symptoms.

Epidemiological studies have demonstrated that Epstein–Barr virus (EBV) is an important aetiological factor for NPC (Henle and Henle, 1985). Serological surveys have found that NPC is highly associated with raised titres for a series of EBV antibodies, high titres of immunoglobulin A (IgA) antibodies to the viral capsid antigen (VCA) and early antigen (EA) of the virus. Previous research has shown that these serological markers are antecedent to the biological onset of tumour cells (Ho, 1978; Henle and Henle, 1985). Using the serological markers, some screening regimes have been launched to identify these high risk groups so as to detect more cases at an early stage of the disease. Table 1 shows the comparison of the stage distribution between screened (for IgA antibodies) and non-screened populations, from the published literature (Zeng et al, 1979, 1980; Zeng, 1985; Zong et al, 1992). The proportion of stage I and II tumours in the screened group is higher (68.7%) than in the non-screened (25%) group. In view of the above, a reasonable screening regime might be to test a population relatively infrequently for the IgA marker. Those testing positively might then be subject to clinical examination, for example indirect mirror examination, at more frequent intervals and advised to consult a doctor after nose-bleeding episodes. It should be noted that although indirect mirror examination is a specialist technique, it may be a reasonable use of resources in this context, in that it is only used on the minority of subjects who test positive on the serological marker.

Although the screening programmes referred to above have indicated that screening can reduce the proportion of advanced stage NPC, the natural history of NPC has not yet been fully investigated. Suppose the disease develops by the following process:

1. All individuals begin free of disease.
2. Some individuals enter a specific EBV reactivation state, manifested by the IgA antibody response to the VCA, putting these individuals at very high risk of developing NPC.
3. Some of the IgA-positive individuals then enter the preclinical but screen-detectable phase (PCDP) of NPC, i.e. to NPC which is asymptomatic but detectable by indirect mirror examination or endoscopy.
4. The individuals with preclinical NPC then advance at an unknown rate to the symptomatic clinical phase.
5. A proportion of the clinical cases finally succumb to death from NPC.

The duration from EBV infection to onset of preclinical NPC is hereafter defined as the incubation period, and the period between entry to the screen-detectable phase and entry to the clinical phase is referred to as the sojourn time. For brevity, we shall refer to the

*Received*
*Revised 7 August 1998*
*Accepted 24 August 1998*

*Correspondence to:* SW Duffy, MRC Biostatistics Unit, Institute of Public Health, University Forvie Site, Robinson Way, Cambridge, CB2 2SR, UK

**Table 1** The comparison of the stage distribution between screen-detected and clinically detected based on published data on NPC screening during 1979–1992

| | Stage I | Stage II | Stage III | Stage IV |
|---|---|---|---|---|
| Screen-detected | 43.2% | 25.5% | 24.1% | 7.2% |
| Clinically detected | 8.4% | 16.6% | 46.8% | 28.1% |

EBV reactivation state simply as EBV infection (although strictly speaking considerably more subjects are EBV infected than those with the relevant antibody response). It is important for evaluation of the efficacy of screening to estimate the rates of transition between the above five states. As the tumour is occult before the clinical phase, empirical data give at best incomplete information on the natural history from EBV infection, biological onset of tumour cells and surfacing to the clinical phase.

In addition, as few studies have reported mortality as the primary end point and no randomized trials of screening have been performed, the efficacy of NPC screening still requires definite confirmation. In 1987, the World Health Organization (WHO) called for a population-based randomized trial to evaluate the efficacy of NPC screening based on mortality from the disease; this has not yet been undertaken. The greatest difficulties in evaluating the efficacy of NPC screening from mortality data are that a long follow-up period or large sample size are required to achieve sufficient statistical power. Current knowledge does not immediately suggest an appropriate length of follow-up or sample size. It would be inadvisable to initiate another NPC screening regime without prior information on the likely mortality as well as a pre-determined sample size based on empirical data and a realistic model. Quantifying the optimum interval between serum screening or between clinical examinations are other difficult design issues to be resolved; determination of the intervals is dependent on the length of the incubation period of EBV infection and on the preclinical screen-detectable period of NPC.

An important strategy in reducing the duration of follow-up required is the use of surrogate end points. It is conceivable that staging of NPC based on the TNM system is a good surrogate end point for deaths from NPC. It is relatively straightforward to estimate predicted deaths based on stage, using previously observed empirical survival data. Even if the surrogate measures are not to be used for the main analyses, they can be used as an aid to design.

In practice we often do not know the exact time of entering a given phase. If a subject tests seropositive for EBV at a given time, we only know that the subject entered the EBV infection phase at some time before then. A multi-state Markov chain model may be used to model the natural history of NPC in terms of the five stages listed above (and described more fully under Materials and Methods). A Markov chain is a process in time which can take a number of states (in the current example, no disease, EBV infection but no NPC, preclinical NPC, clinical NPC) and individuals move from state to state at random points in time but with rates of transition that are estimable from empirical data (Chen et al, 1997). The major assumption is that if an individual is known to be in a given state, for example preclinical NPC, at time t, knowledge of that individual's history before time t does not add to our prediction of what will happen to that individual thereafter. The advantage of using a Markov chain model is the ability to estimate the rate of EBV infection and onset of preclinical cancer even if the exact times to such events are not known.

The aims of this study are to:

1. derive rough estimates of the rate of EBV infection – transition rates from EBV infection to preclinical cancer (the incubation period) – and from the screen-detectable phase to the clinical phase, based on a multi-state Markov chain model;
2. compare the estimated stage distribution of NPC by different screening frequencies (including an unscreened group) based on (1);
3. predict mortality based on (2) and on published survival rates by stage;
4. calculate the required sample size or duration of follow-up using either NPC mortality or a surrogate for mortality in a hypothetical randomized trial of NPC screening.

The above strategy illustrates the use of the models on published data, not to obtain definitive answers to questions of the efficacy of screening, but to derive approximate prior estimates to assist in the design of future studies to obtain such answers.

### MATERIALS AND METHODS

### Definition of the Markov chain model

In order to depict the natural history of NPC a four-state Markov model is proposed including No disease (0), EBV infection (1), preclinical screen-detectable phase (2) and clinical phase (3). We assume that this model is progressive, i.e. no regression from EBV infection to no EBV infection is possible, with a similar assumption for more serious states. There are three parameters in this model, $\lambda_1$, $\lambda_2$, $\lambda_3$, representing the rate of EBV infection, the transition rate from EBV infection to the PCDP and the transition rate from the PCDP to the clinical phase respectively. This is expressed as the following transition matrix:

$$\begin{array}{l} \\ \\ \textbf{No disease} \\ \textbf{EBV infection} \\ \textbf{PCDP NPC} \\ \textbf{Clinical NPC} \end{array} \begin{array}{cccc} \textbf{No} & \textbf{EBV} & \textbf{PCDP} & \textbf{Clinical} \\ \textbf{disease} & \textbf{infection} & \textbf{NPC} & \textbf{NPC} \end{array} \\ \begin{bmatrix} -\lambda_1 & \lambda_1 & 0 & 0 \\ 0 & -\lambda_2 & \lambda_2 & 0 \\ 0 & 0 & -\lambda_3 & \lambda_3 \\ 0 & 0 & 0 & 0 \end{bmatrix} \quad (1)$$

Based on (1), the transition probabilities for building up the likelihood function can be derived, as shown in Appendix 1.

### Data for the development of likelihood function

Table 2 shows different transition histories and numbers of transitions of each type for the studies outlined in Table 1. The application of the transition probabilities in Appendix 1 to the corresponding transitions in Table 2 enables us to develop the likelihood function for each study to estimate the three parameters. For example, Zeng et al (1985) found 1118 EBV infections among 20 276 attendants and 18 asymptomatic NPC cases by further indirect mirror examination. The corresponding transition probabilities for no disease, EBV infection and asymptomatic NPC during time t, say period to age at first screen, are denoted $p_{00}(t)$, $p_{01}(t)$, and $p_{02}(t)$. The likelihood function based on this can be written as

$$L(.) = [p_{00}(t)]^{19590} \times [p_{01}(t)]^{1118} \times [p_{02}(t)]^{18} \quad (2)$$

© Cancer Research Campaign 1999

**Table 2** Numbers of possible types of transition for NPC based on Markov model (1) by studies from 1980 to 1992

| Studies | Types of transition: No disease → No disease (0 → 0) | No disease → EBV infection (0 → 1) | No disease → Preclinical NPC (0 → 2) | No disease → Clinical NPC (0 → 3) |
|---|---|---|---|---|
| Zeng et al (1985) | 19 590 | 1118 | 18 | – |
| Zeng (1985) | 15 173 | 144 | 7 | – |
| Zong et al (1992) | (a) Screened: | | | |
| | 39 225 | 2792 | 31 | – |
| | (b) Control: | | | |
| | 397 000 | – | – | 140 |
| Zeng et al (1979, 1980) | 144 496 | 405 | 55 | – |

The likelihood function for other studies may be developed in a similar way. It should be noted that, although the transition from the PCDP to clinical NPC is not directly observed, the information on transitions from no disease to clinical NPC from Zong et al (1992) allows us to estimate this parameter, $\lambda_3$.

The method used for estimation of the parameters was a quasi-likelihood approach equating the observed numbers of transitions with the expected, using a non-linear regression model. Details of this method are given by Duffy et al (1995) and Chen et al (1996). Since the data used in this study were from published accounts of various studies, we estimated the transition parameters for each study separately, then calculated weighted averages over all studies, weighting for each study by the numbers of participants. The pooled estimates were then used to predict cases and deaths from NPC.

The transition probabilities from equation (2) were used to calculate predicted EBV-positive and NPC cases detected by screen (screen-detected) or diagnosed between screens (interval cancers) for various screening regimes.

### RESULTS

### Parameter estimation

Table 3 shows estimated results for three parameters, $\lambda_1$ (no disease to EBV infection), $\lambda_2$ (EBV infection to PCDP) and $\lambda_3$ (PCDP to clinical phase), for each study. The estimated weighted average instantaneous transition rates were 0.00075, 0.002819 and 0.32583 for $\lambda_1$, $\lambda_2$ and $\lambda_3$ respectively. Since only one study in Table 2 provided data on symptomatic NPC, the estimate of $\lambda_3$ is based on this study only (Zong et al, 1992).

The parameter estimates in Table 3 indicate an annual rate of EBV infection of just under 1 per 1000, and an annual rate of progression to preclinical NPC in EBV-positive subjects of around 3 per 1000. The inverse of $\lambda_3$ in Table 3 estimated the mean sojourn time (the average time period spent in the preclinical phase) as approximately 3.1 years.

### Application: prediction of NPC cases and deaths based on the incidence rate in Hong Kong

We have estimated the parameters for transition from no disease to EBV infection, from EBV infection to the PCDP and from the PCDP to the clinical phase. We apply these estimates to calculate the predicted number of EBV infections, preclinical NPC cases detected by screening PCDP and clinical NPC cases for a hypothetically screened population from Hong Kong.

Since the EBV infection rate obtained from the present study was not based on the Hong Kong population, the underlying EBV infection rate is adjusted to yield NPC rates that are representative of the Hong Kong population. Since no studies have so far reported the exact underlying EBV infection rate for Hong Kong we used an indirect method to adapt the underlying rate. We first calculate an age-specific cumulative incidence rate based on the present parameters, and adjusted $\lambda_1$ to give the corresponding cumulative incidence rate for Hong Kong calculated using data from Cancer Incidence in Five Continents (Muir et al, 1987). We use the male population, which has a particularly high incidence of NPC. The prevalence of EBV infection was estimated as 17.218% in Hong Kong males. The estimated incidence rate of NPC in the age group 40–69 was 74.4 per 100 000, which is consistent with the figure of 76.6 per 100 000 from Cancer Incidence in Five Continents.

We present predicted results for four screening regimes in a hypothetical population of 100 000 Hong Kong Chinese males. The four regimes are:

1. Three-yearly IgA/VCA plus annual indirect mirror examination.
2. Three-yearly IgA/VCA plus 3-yearly indirect mirror examination.
3. Six-yearly IgA/VCA plus annual indirect mirror examination.
4. Six-yearly IgA/VCA plus 3-yearly indirect mirror examination.

**Table 3** Estimated parameters for NPC based on Markov model (1)

| Studies | $\lambda_1$: No disease → EBV infection (0 → 1) | $\lambda_2$: EBV infection → Preclinical NPC (1 → 2) | $\lambda_3$: Preclinical NPC → Clinical NPC (2 → 3) |
|---|---|---|---|
| Zeng et al (1985) | 0.0009394 | 0.0005288 | – |
| Zeng (1985) | 0.00018 | 0.00173 | – |
| Zong et al (1992) | 0.00117 | 0.0051 | 0.3258 |
| Zeng et al (1979, 1980) | 0.00043 | 0.0005697 | – |
| Weighted average | 0.00075 | 0.002819 | 0.3258 |

 *© Cancer Research Campaign 1999*

**Table 4** Predicted numbers of IgA positive cases and nasopharyngeal carcinoma in males by annual and 3-yearly NPC screening regimes in conjunction with 3-yearly and 6-yearly IgA screening programmes based on the Hong Kong incidence rate with *n* = 100 000, and assuming 100% sensitivity for IgA screening

| | Three-year IgA screening Clinical assessment | | Six-year IgA screening Clinical assessment | |
|---|---|---|---|---|
| **Diagnostic group** | **One-year** | **Three-year** | **One-year** | **Three-year** |
| Total serum IgA (+) | 19020.9 | 19020.9 | 19013.1 | 19013.1 |
| Total screen-detected NPC | 400.5 | 336.6 | 397.1 | 334.9 |
| Interval cancers after negative Serum IgA | 2.0 | 2.0 | 6.5 | 6.5 |
| Interval cancers after negative clinical assessment | 43.5 | 107.4 | 42.3 | 104.6 |
| Total interval cancers | 45.5 | 109.4 | 48.9 | 111.2 |
| Total NPC cases | 446 | 446 | 446 | 446 |

Table 4 shows the predicted EBV-positive and NPC cases for the four screening regimes. The proportion of interval NPC cases expected for screening regimes (1) three-yearly IgA/VCA plus annual indirect mirror examination, and (2) three-yearly IgA/VCA plus three-yearly indirect mirror examination, were 0.1 and 0.25 respectively. This yielded 59% [0.25–0.1]/0.25) for the prevented fraction of interval NPC cases due to changing from 3-yearly to 1-yearly indirect mirror examination under 3-yearly IgA/VCA. As the difference between 3-yearly and 6-yearly IgA/VCA was not substantial, a similar prevented fraction was observed for screening regime (3) and (4), which was based on 6-yearly screening for IgA/VCA.

From the results in Table 4, we use the proportions in Table 1 of screen-detected and clinically detected tumours by stage to predict the incidence by stage. Stage-specific 5-year survival is given by published literature (Sham and Choy, 1990; Sasco, 1991) as 80%, 69%, 42% and 15% for stages I to stage IV respectively. These were then applied to the stage distributions to give expected 5-year deaths from NPC by screening regime. Results are shown in Table 5 for 3-yearly and 6-yearly serological testing.

With 3-yearly serum screening, one would expect annual indirect mirror examination to reduce 5-year mortality by 33%, and 3-yearly indirect mirror examination to lead to a 28% reduction. With 6-year serum screening, the results are almost exactly the same. Thus, the frequency of indirect mirror examinations between serum screens has a greater bearing on the expected mortality than the frequency of serum testing.

### DISCUSSION

In this study, we have estimated 33% and 28% reductions in mortality from NPC attributed to intensive indirect mirror examination of IgA/VCA-positive subjects for annual vs no screening, and 3-yearly vs no screening, by applying a Markov chain model to the published results for NPC mass screening to estimate the relevant parameters. The difference in the proportion of screen-detected and clinically detected cancers between 3-yearly and 6-yearly IgA/VCA screening was not substantial. This suggests that 6-yearly serological marker screening plus annual indirect mirror examination can reduce the mortality from NPC by approximately 30%. From the cost-effectiveness viewpoint, it might be argued that 6-yearly IgA/VCA for NPC, in combination with 3-yearly indirect mirror examination, might be sufficient.

While these results cannot be regarded as strong evidence of a mortality benefit, they give a useful estimate of the size of likely benefit and indicate the regime of choice if a genuine trial were proposed. While the published literature does not give data in sufficient detail to assess the fit of our models, the results suggest reasonable design strategies for a population-based trial of NPC screening. Such a trial, however, in addition to the primary purpose of evaluating the effect of the screening, would also provide diagnostics for the models.

To simplify the calculations, we estimated the distribution of stage from previously published stage distributions, rather than directly estimating transition rates between stages. It could be argued that one can use a six-state Markov chain model to estimate these parameters, as used by Chen et al (1997) for modelling breast cancer progression. The major resource available for such estimation is the stage data from prevalence screening in the published literature, as shown in Table 6. The detailed algebra and calculations to estimate progression rates from these data are given in Appendix 2.

**Table 5** Stage distribution and predicted 5-year deaths from NPC for indirect mirror examination frequencies of 1 year and 3 years, under 3-yearly and 6-yearly serum IgA testing, and for no screening at all

| Serum IgA testing frequency | Indirect mirror examination | Outcome | Stage I | Stage II | Stage III | Stage IV | Total |
|---|---|---|---|---|---|---|---|
| 3-yearly | 3-yearly | Cancers | 155 | 104 | 132 | 55 | 446 |
| | | Deaths | 31 | 32 | 77 | 47 | 187 |
| | 1-yearly | Cancers | 177 | 110 | 118 | 41 | 446 |
| | | Deaths | 35 | 34 | 68 | 35 | 172 |
| | No screening | Cancers | 38 | 74 | 209 | 125 | 446 |
| | | Deaths | 8 | 23 | 121 | 106 | 258 |
| 6-yearly | 3-yearly | Cancers | 154 | 104 | 133 | 55 | 446 |
| | | Deaths | 31 | 32 | 77 | 47 | 187 |
| | 1-yearly | Cancers | 176 | 109 | 119 | 42 | 446 |
| | | Deaths | 35 | 34 | 69 | 36 | 174 |
| | No screening | Cancers | 38 | 74 | 209 | 125 | 446 |
| | | Deaths | 8 | 23 | 121 | 106 | 258 |

*© Cancer Research Campaign 1999*

**Table 6** Numbers of possible types of stage transition for NPC based on Markov model (2) by studies from 1980 to 1992

| Studies | Types of transition: No disease → no disease (0 → 0) | No disease → Stage I (0 → 1) | No disease → Stage II (0 → 2) | No disease → Stage III+ (0 → 3) |
|---|---|---|---|---|
| Zeng et al (1979, 1980) | 147 974 | 12 | 19 | 23 |
| Zeng et al (1985) | 20 708 | 10 | 6 | 2 |
| Zong et al (1992) | 42 007 | 28 | 5 | 8 |

**Table 7** Stage distribution and calculation of variance components for mortality and surrogate endpoint by different screening regime

| Stage | 1-year clinical assessment ($q_{i1}$) | 3-yearly clinical assessment ($q_{i3}$) | No clinical assessment ($q_{i0}$) | 5-year death rate ($P_i$) |
|---|---|---|---|---|
| I | 0.3964 | 0.3466 | 0.0843 | 0.20 |
| II | 0.2462 | 0.3333 | 0.1664 | 0.31 |
| II | 0.3964 | 0.2964 | 0.4683 | 0.58 |
| IV | 0.0933 | 0.1231 | 0.2810 | 0.85 |
| $1/\Sigma Pq$ | 2.57 | 2.38 | 1.73 | |
| $\Sigma P^2 q/(\Sigma Pq)^2$ | 1.30 | 1.28 | 1.14 | |

Using the methods in Appendix 2, the transition rates from stage I to stage II and from stage II to stage III+ within the PCDP were estimated as 0.06055 and 0.05054. Transition rates from preclinical stage I to clinical stage I, and preclinical stage II+ to clinical stage II+, were estimated as 0.011445 and 1.72 respectively. Based on these parameters, we calculated the expected proportion of stage I cancers by different detection modes. Detailed calculations are given in Appendix 3. For the prevalent screen, we estimated that approximately 45% of cases would be stage I tumours. This is very close to the figure for screen-detected cases in Table 1 based on the published literature. For cases after negative IgA screening, a 6-year regime of IgA screening gave 16% stage I clinical cases during 6 years. This is consistent with the figure for clinically detected cases in Table 1. For incident screens, annual and 3-yearly screening regimes of clinical assessment yielded 98% and 94% of screen-detected cases being in stage I. For interval cancers, the estimated proportions of stage I tumours for annual and 3-yearly clinical assessment were 37% and 23% respectively.

The predicted mortality estimated in this study can be applied to calculate power for a randomized trial that might be launched in the future. The use of surrogate end points for deaths from NPC, such as stage distribution, could also be considered. We calculated the required sample size for surrogate end points based on Day and Duffy's method (1996) which was used in their paper to calculate sample size for different frequencies of breast cancer screening. The formulae for the power calculations for both a mortality end point and a surrogate end point are shown in Appendix 4.

To calculate power for a possible trial in Hong Kong, we use the estimates of 33% and 28% reduction in mortality from NPC attributed to screening for annual and 3-yearly regimes as compared to no screening. These come from the pragmatic estimates using the stage distributions from the literature and the simple four-state model in equation (1), in view of the fact that these are largely in agreement with estimates from the more sophisticated and complex models of Appendices 2 and 3. We assume two serum IgA tests 6 years apart, with annual or 3-yearly clinical assessment of those screened IgA-positive, i.e. a 6-year screening phase of the trial, with seven or three rounds of clinical assessment. Suppose each arm has 50 000 subjects with 76.6 per 100 000 incidence rate, that of Hong Kong males aged 40–69. Assume an average of 5 years follow-up for survival of the NPC cases. The variances for mortality and surrogate end point for annual screening versus no screening were calculated as 0.0187 and 0.0083, respectively, according to Table 7 and expression (A-5) and (A-6) in Appendix 4. With 50 000 subjects on each arm and two-sided significance testing at 5% level, a trial based on actual mortality would have 82% power for a comparison of annual clinical examination with no screening, and 69% power for a comparison of 3-yearly clinical examination with no screening. The corresponding power estimates using the surrogate end point of predicted deaths from stage would be 99% and 89%.

Since there is, as yet, no randomized trial evidence on the effect of screening for NPC, it is arguably necessary for a future trial to be based on actual mortality. For such a trial to have high sensitivity, very large sample sizes are needed. With annual clinical examination, a 5% significance level and two-sided testing, 61 000 persons per arm will be required for 90% power. With 3-yearly clinical examination, 87 000 per arm would be needed.

In conclusion, a Markov chain model was developed, based on limited published data, to estimate relevant parameters to predict the mortality reduction to be expected by screening for NPC. The application of these parameters to a population with a relatively high incidence rate yields support for the efficacy of 6-yearly serologic screening followed by more frequent clinical assessment (indirect mirror examination) of those who are seropositive. Annual and 3-yearly indirect examination might be expected to reduce the number of deaths from NPC by 33% and 28%. Prediction of deaths from these models can aid design of a future randomized trial in a high incidence area such as Hong Kong.

 © Cancer Research Campaign 1999

### ACKNOWLEDGEMENTS

We thank Professor KK Cheng of the University of Birmingham for helpful discussion. We also thank the Editor and an anonymous referee for improvements to the paper.

### REFERENCES

Chen GJ, Chen JY, Hsu MM, Hsu MM, Liu MY, Cho SM, Hsu MM, Lynn TC, Shieh J, Tu SM, Beasley RP, Hwang LY, Lee HH, Kuo SL and Yang CS (1988) Epidemiological characteristics and early detection of nasopharyngeal carcinoma in Taiwan. In *Head and Neck Oncology Research*, Wolf GT and Carey TE (eds), pp. 505–513. Kugler: Amsterdam

Chen HH, Duffy SW and Tabar L (1996) A Markov chain method to estimate the tumour progression rate from preclinical to clinical phase, sensitivity and positive predictive value for mammography in breast cancer screening. *The Statistician* **45**: 307–317

Chen HH, Duffy SW, Tabar L and Day NE (1997) Markov chain models for progression of breast cancer. Part 1: tumour attributes and the preclinical screen-detectable phase. *J Epidemiol Biostat* **2**: 9–23

Day NE and Duffy SW (1996) Trial design based on surrogate endpoints: application to comparison of different breast screening frequencies. *J R Statist Soc A* **159**: 49–60

Duffy SW, Chen HH, Tabar L and Day NE (1995) Estimation of mean sojourn time in breast cancer screening using a Markov chain model of both entry to and exit from the preclinical detectable phase. *Statist Med* **14**: 1531–1543

Henle W and Henle G (1985) Epstein–Barr virus and human malignancies. *Adv Viral Oncol* **5**: 201–238

Ho JHC (1978) Stage classification of nasopharyngeal cancer: a review. In *Nasopharyngeal Carcinoma*: *Etiology and Control*, de The G and Ito Y (eds), pp. 99–113. International Agency for Research on Cancer: Lyon

Lanier A, Bender T, Talbot M, Wilmeth S, Tschopp C, Henle W, Henle G, Ritter D and Terasaki P (1980) Nasopharyngeal carcinoma in Alaskan Eskimos, Indians and Aleuts: a review of cases and study of Epstein–Barr virus, HLA and environmental risk factors. *Cancer* **46**: 2100–2106

Lee HP, Day NE and Shanmugaratnam K (1988) *Trends in Cancer Incidence in Singapore 1968–1982*. International Agency for Research on Cancer: Lyon

Muir C, Waterhouse J, Mack T, Powell J and Whelan S (1987) *Cancer Incidence in Five Continents, Vol V*. International Agency for Research on Cancer: Lyon

Sasco AJ (1991) Screening for nasopharyngeal carcinoma. In *Cancer Screening*, Miller AB, Chamberlain J, Day NE, Hakama M and Prorok PC (eds), pp. 375–387. Cambridge University Press: Cambridge

Sham JST and Choy D (1990) Prognostic factors of nasopharyngeal carcinoma: a review of 759 patients. *Br J Radiol* **63**: 51–58

Zeng Y (1985) Seroepidemiological studies on nasopharyngeal carcinoma in China. *Adv Cancer Res* **43**: 121–138

Zeng Y, Liu YX, Liu CR, Chen S, Wei J, Zhu J and Zai H (1979) Application of immunoenzymatic method and immunoautoradiographic method for the mass survey of nasopharyngeal carcinoma. *Chinese J Oncol* **1**: 2–7

Zeng Y, Liu YX, Liu CR, Chen S, Wei J, Zhu J and Zai H (1980) Application of an immunoenzymatic method and immunoautoradiographic method for a mass survey of nasopharyngeal carcinoma. *Intervirology* **13**: 162–168

Zeng Y, Zhang LG, Wu YC, Huang YS, Huang NQ, Li JY, Wang YB, Jiang MK, Fang Z and Meng NN (1985) Prospective studies on nasopharyngeal carcinoma in Epstein–Barr virus IgA/VCA antibody positive persons in Wuzhou City, China. *Int J Cancer* **36**: 545–547

Zong YS, Sham JST, Ng MH, Ou XT, Guo YQ, Zheng SA, Liang JS and Hong Q (1992) Immunoglobulin A against viral capsid antigen of Epstein–Barr virus and indirect mirror examination of the nasopharynx in the detection of asymptomatic nasopharyngeal carcinoma. *Cancer* **69**: 3–7

### Appendix 1

The formulae for transition probabilities for expression (2) in text are:

$$P_{00} = e^{-\lambda_1 t}$$

$$P_{01} = \int_0^t \lambda_1 e^{-\lambda_1 s} e^{-\lambda_2 (t-s)} ds$$

$$P_{02} = \int_0^t \lambda_1 e^{-\lambda_1 s} \int_0^{t-s} \lambda_2 e^{-\lambda_2 u} e^{-\lambda_3 (t-s-u)} duds$$

$$P_{03} = \int_0^t \lambda_1 e^{-\lambda_1 s} \int_0^{t-s} \lambda_2 e^{-\lambda_2 u} \int_0^{t-s-u} \lambda_3 e^{-\lambda_3 r} drduds$$

$$P_{11} = e^{-\lambda_2 t}$$

$$P_{12} = \int_0^t \lambda_2 e^{-\lambda_2 s} e^{-\lambda_3 (t-s)} ds$$

$$P_{13} = \int_0^t \lambda_2 e^{-\lambda_2 s} \int_0^{t-s} \lambda_3 e^{-\lambda_3 u} duds$$

$$P_{22} = e^{-\lambda_3 t}$$

$$P_{23} = 1 - e^{-\lambda_3 t} \qquad \text{(A-1)}$$

### Appendix 2

The transition matrix for a stage model would be:

| | No disease | EBV Infection | Preclinical Stage I | Preclinical Stage II+ | Clinical Stage I | Clinical Stage II+ |
|---|---|---|---|---|---|---|
| **No disease** | $-\gamma_1$ | $\gamma_1$ | 0 | 0 | 0 | 0 |
| **EBV infection** | 0 | $-\gamma_2$ | $\gamma_2$ | 0 | 0 | 0 |
| **Preclinical Stage I** | 0 | 0 | $-(\gamma_3 + \gamma_4)$ | $\gamma_3$ | $\gamma_4$ | 0 |
| **Preclinical Stage II+** | 0 | 0 | 0 | $-\gamma_5$ | 0 | $\gamma_5$ |
| **Clinical Stage I** | 0 | 0 | 0 | 0 | 0 | 0 |
| **Clinical Stage II+** | 0 | 0 | 0 | 0 | 0 | 0 |

Estimation of parameter $\gamma_4$ and $\gamma_5$ in text was performed by first estimating the transition rates for progression in the more complex model involving preclinical disease at stages I, II and III+. We therefore estimated the following transition rates:

$\mu_1$: Preclinical stage I to preclinical stage II  
$\mu_2$: Preclinical stage II to preclinical stage III+  
$v_1$: Preclinical stage I to clinical stage I  
$v_2$: Preclinical stage II to clinical stage II  
$v_3$: Preclinical stage III+ to clinical stage III+.

Using the methods of Chen et al (1997) and the data in Table 6, $\mu_1$ and $\mu_2$ were estimated as 0.06055 and 0.05054. In conjunction with sojourn time and the proportion of clinical stage 1 tumours, $v_1$ was back-calculated by the following:

$$E(p = clinicalstage1) = \frac{\int_0^t \nu_1 e^{-\nu_1 s} e^{-\mu_1 s} ds}{\int_0^t \lambda_3 e^{-\lambda_3 s}} = 0.08 \qquad \text{(A-2)}$$

© Cancer Research Campaign 1999 

Substitution of $\mu_1$ = 0.06055 and $\lambda_3$ = 0.32583 (Table 3) and t = 3.1 years give $v_1$ as 0.011445.

Similarly, the estimate of $v_2$ was back-calculated by the following expression:

$$E\,(p_2 = \textit{clinical stage } 2) = \frac{\int_0^t \mu_1 e^{-\mu_1 s} e^{-v_2 s} \int_0^{t-s} v_2 e^{-v_2 u} e^{-\mu_2 u} du ds}{\int_0^t \lambda_3 e^{-\lambda_3 s}} = 0.093 \tag{A-3}$$

Substitution as for (A-2) and $\mu_2$ = 0.0504 gives $v_2$ as 0.31291.

As the proportion of stage II+ cancers among clinical cases is high, the mean duration from preclinical to clinical stage II+, i.e. 1/ $v_3$, must be very short, and can be regarded as approximately zero in years. Instead of using stage II and stage III+, we calculate the expected time spent in preclinical stage II+ before moving to the clinical phase as the weighted average of the times to clinical disease from preclinical stage II and III+ to clinical stage II and III+, according to the proportion of clinical tumours in stage II and III+ from Table 1. We then invert the expected length of time in preclinical stage II+ to give the transition rate to clinical disease as:

$(1/v_2\ (16.64/91.57)+0(74.93/91.57))^{-1} = 1.72$

### Appendix 3

Estimation of the proportion of stage I tumours by detection mode is as follows:

### *(1) Prevalent screen*

Given the parameters in Appendix 2, the proportion of stage I tumours at the prevalent screen for a population 50 years of age on average was calculated according to the following expression:

$$P_1 = \frac{\int_0^{50} \gamma_1 e^{-\gamma_1 s} \int_0^{50-t-s} \gamma_2 e^{-\gamma_2 s} e^{-(\gamma_3+\gamma_4)\times(50-t-s)} dsdt}{\int_0^{50} \gamma_1 e^{-\gamma_1 s} \int_0^{50-s} \gamma_2 e^{-\gamma_2 s} e^{-\gamma_4 \times (50-t-s)} dsdt} = 0.4510 \tag{A-4}$$

This yields 45.10% stage I among prevalent NPC cancers.

### *(2) Incident screen*

A similar calculation was performed to estimate the proportion of stage I cancers for incident screens by substituting screening interval for age.

### *(3) Interval cancers*

The expression for the proportion of stage 1 cases among interval cancers is:

$$I_1 = \frac{\int_0^{50} \gamma_1 e^{-\gamma_1 t} \int_0^{50-t} \gamma_2 e^{-\gamma_2 s} \int_0^{50-t-s} \gamma_4 e^{-\gamma_4 u} e^{-\gamma_3 u} dudsdt}{\int_0^{50} \gamma_1 e^{-\gamma_1 t} \int_0^{50-t} \gamma_2 e^{-\gamma_2 s} \int_0^{50-t-s} \gamma_4 e^{-\gamma_4 u} e^{-\gamma_3 u} dudsdt + \int_0^{50} \gamma_1 e^{-\gamma_1 s} \int_0^{50-s} \gamma_2 e^{-\gamma_2 s} \int_0^{50-t-s} \gamma_3 e^{-\gamma_3 u} \int_0^{50-t-s-u} \gamma_5\, e^{-\gamma_5 V} dvdudsdt}$$

### Appendix 4

Power calculations can be performed as follows.

For annual screening versus no screening, for example, suppose screening was annually applied to a hypothetical population with an incidence rate of NPC of 76.6 per 100 000 for 6 years. According to Day and Duffy's method (1996), the variance for the mortality end point is:

$$V_{obs} = \frac{1}{N \times 0.000766 \times 6}\left(\frac{1}{\sum P_i q_{i1}} + \frac{1}{\sum P_i q_{i0}}\right) \tag{A-5}$$

and for the surrogate endpoint, the variance is

$$V_{pred} = \frac{1}{N \times 0.000766 \times 6}\left(\frac{\sum P^2{}_i q_{i1}}{(\sum P_i q_{i1})^2} + \frac{\sum P^2{}_i q_{i0}}{(\sum P_i q_{i0})^2}\right) \tag{A-6}$$

where $P_i$ is the probability of death from NPC for an individual in category i of the surrogate end point (stage in our case); $q_{i1}$ and $q_{i0}$ are the probability of being in category i in each arm j, j = 0 (no screening) and 1 (annual screening); and N is the number of individuals on each arm of the trial.

Suppose the estimated reduction in mortality from NPC was estimated as $T_1/T_0$, where $T_1 = \Sigma P_i q_{i1}$ and $T_2 = \Sigma P_i q_{qi0}$. Power, denoted by 1-β, for a test at significance level α (two-sided), can be calculated as:

$1\text{-}\beta = \varphi\,(-Z\text{-}\log(T_1/T_0)/S)$

where Z is the upper (1–0.5α) point of the standard normal distribution, φ is the standard normal distribution function and S is the square root of $V_{obs}$ or $V_{pred}$, depending on whether observed mortality or that predicted from the surrogate is to be used in the analysis.

 *© Cancer Research Campaign 1999*
